# A Scientometric Review of Infant Cry and Caregiver Responsiveness: Literature Trends and Research Gaps over 60 Years of Developmental Study

**DOI:** 10.3390/children10061042

**Published:** 2023-06-10

**Authors:** Alessandro Carollo, Pietro Montefalcone, Marc H. Bornstein, Gianluca Esposito

**Affiliations:** 1 Department of Psychology and Cognitive Science, University of Trento, Corso Angelo Bettini 31, 38068 Rovereto, Italy; alessandro.carollo@unitn.it (A.C.); pietro.montefalcone@studenti.unitn.it (P.M.); 2 Eunice Kennedy Shriver National Institute of Child Health and Human Development, Bethesda, MD 20892, USA; marc.h.bornstein@gmail.com; 3 United Nations Children’s Fund, New York, NY 10017, USA; 4 Institute for Fiscal Studies, London WC1E 7AE, UK

**Keywords:** infant cry, parental sensitivity, caregiver responsiveness, infant vocalizations, CiteSpace, scientometrics, cry responsiveness

## Abstract

Infant cry is an adaptive signal of distress that elicits timely and mostly appropriate caring behaviors. Caregivers are typically able to decode the meaning of the cry and respond appropriately, but maladaptive caregiver responses are common and, in the worst cases, can lead to harmful events. To tackle the importance of studying cry patterns and caregivers’ responses, this review aims to identify key documents and thematic trends in the literature as well as existing research gaps. To do so, we conducted a scientometric review of 723 documents downloaded from Scopus and performed a document co-citation analysis. The most impactful publication was authored by Barr in 1990, which describes typical developmental patterns of infant cry. Six major research thematic clusters emerged from the analysis of the literature. Clusters were renamed “Neonatal Pain Analyzer” (average year of publication = 2002), “Abusive Head Trauma” (average year of publication = 2007), “Oxytocin” (average year of publication = 2009), “Antecedents of Maternal Sensitivity” (average year of publication = 2010), “Neurobiology of Parental Responses” (average year of publication = 2011), and “Hormonal Changes & Cry Responsiveness” (average year of publication = 2016). Research clusters are discussed on the basis of a qualitative inspection of the manuscripts. Current trends in research focus on the neurobiology of caregiver responses and the identification of factors promoting maternal sensitivity. Recent studies have also developed evidence-based strategies for calming crying babies and preventing caregivers’ maladaptive responses. From the clusters, two topics conspicuously call for future research: fathers’ responsiveness to infant cry and the impact of caregiver relationship quality on cry responsiveness.

## 1. Introduction

In mammalian species, newborns use visual and acoustic signals to communicate different needs [1,2]. Across phylogenesis, infants’ distress vocalizations have proven useful in increasing the survival of the newborns by eliciting a fixed set of caregiving responses [1,3,4,5]. Caregivers and infants share a synchronous attuned relationship, where coordinated behavioral and physiological responses in both members of the dyad prompt caregivers to provide optimal caregiving [6,7]. In response to their infant crying, mothers, as compared to non-mothers, show enhanced neural activity in brain regions typically associated with emotional processing. This finding indicates that infant crying is perceived by mothers as an emotionally significant signal that requires their attention [1,8]. Response to infant crying also differs between mothers and fathers at a physiological level. When exposed to their infants’ crying, mothers’ heart rate decelerates, followed by a quick acceleration; conversely, fathers exhibit only a decrease in heart rate. These results suggest that, when exposed to infant crying, mothers ready themselves for potential intervention, whereas fathers tend to demonstrate passive attentiveness [5,9]. Some gender differences in response to crying can also be observed at the neural level. In a functional magnetic resonance imaging study, De Pisapia et al. [10] found that infants’ hunger cries elicit different neural responses in males as compared to females. In males, infant cry is accompanied by neural activity in the dorsal medial prefrontal and posterior cingulate regions, two brain regions involved in mind-wandering during awake rest. However, females show a reduction in activity in these same brain regions. These results suggest that brain activity in females, more than males, is strongly affected by the sounds of infant hunger cries, which tend to interrupt mind-wandering in women.

Cry has also been studied in regard to atypical development. People with atypical development use both conventional and unconventional nonverbal behaviors to communicate. Esposito et al. [11] showed that crying behavior in individuals with autism spectrum disorder (ASD) can be difficult to understand and can negatively affect the quality of care provided by caregivers. The way caregivers perceive crying is influenced by their interpretation of different acoustic characteristics of crying that, in children with ASD, are atypical. Specifically, the length of pauses during crying is a more significant factor in determining negative perceptions of crying episodes than the number of utterances or fundamental frequency [12].

For caregivers, the capacity to understand and attribute the correct meaning to infant cry is fundamental to their establishment of a strong and secure relationship [13,14,15]. This process is possible as distinct patterns in the frequency, duration, and pitch of vocalizations reportedly correspond to distinct infant needs [16,17]. For instance, pain cries, as compared to hunger cries, are characterized by signals with higher frequency [18]. These subtle differences in the acoustic patterns of vocalizations cue caregivers to provide adequate care. In fact, adults seem to be able to discriminate between acoustic features of infant cry [19]. This is especially true for adults who have prolonged experience with infants, such as parents, caregivers, pediatricians, and nurses [16]. For young parents, however, it is often challenging to differentiate between, and correctly interpret, infant cry signals [16]. Unsoothable crying spells render caregivers powerless and guilty. At extremes, overwhelmed parents in some cases might respond violently to unsoothable infants [20,21,22,23]. Physical punishments of children are common worldwide, with higher rates documented in low- and middle-income countries [24]. Abusive head trauma and shaken baby syndrome are terms used to describe physical and psychological consequences due to shaking or hitting an infant or a small child [25,26]. It is therefore of extreme importance for caregivers to develop effective and positive response strategies to infant cry. Attempts in this direction were undertaken by Esposito et al. [27] and Ohmura et al. [28]. Both of these studies suggest that, when mothers try to calm their infant, a carrying strategy is more effective than simple holding. Transporting the baby drastically reduces infant crying and typically favors sleep within 5 min [28].

Given the developmental relevance and health significance of studying typical and atypical caregiver responses to infant crying, the current study aimed to summarize the literature in the field using a scientometric approach. Scientometrics lies at the intersection of scientific mapping (i.e., visualization of the temporal evolution of a research domain) and bibliometric analysis (i.e., application of quantitative techniques to bibliometric data) [29,30,31]. Compared to other review methods, the scientometric approach enables the analysis of a larger sample of data with a data-driven approach. Therefore, it is less prone to human biases in the selection and analysis of source documents. The scientometric approach has been applied previously by Carollo et al. [32] to identify the main themes in the literature on interpersonal synchrony in caregiver–child interactions. However, to our knowledge, no article has yet applied the scientometric approach to review the literature on infant cry and caregiver responsiveness. From analysis of the quantitative relationships among publications in the literature, the current work investigates the most impactful documents as well as the main thematic domains in the field of infant cryingand caregiver responsiveness. In doing so, the current work also identifies research gaps and possible future directions of study.

## 2. Materials and Methods

### 2.1. Data Collection from Scopus

The current scientometric review follows the pipeline of previously published works in the field of social neuroscience, clinical psychology, and addiction neuroscience (e.g., [33,34]).

All publications used for this article were downloaded from Scopus, as it guarantees the widest coverage of indexed journals [35,36]. The search string was: TITLE-ABS-KEY ((child* OR infant* OR neonate* OR newborn* OR baby OR babies OR toddler*) AND (cry OR crying OR “separation call*”) AND (responsiveness OR sensitivity OR “cry* analysis” OR “cry* pattern*” OR “cry* scheme*” OR “cry* profile*”)). The terms of the search string were optimized to include in the sample all relevant literature on infant cry and caregiver responsiveness. In doing so, key terms were selected to reduce the inclusion of non-relevant publications. Moreover, the quality of the identified sample of documents was confirmed through a qualitative inspection of document titles and abstracts. Altogether, 723 documents published from 1962 to 2022 were identified. All data were collected on 17 November 2022. A preliminary analysis of the citing documents was conducted by means of the *bibliometrix* package for R [37]. Scopus data were downloaded in .ris format to run the analysis on CiteSpace and in .bib format to run the analysis with *bibliometrix* package.

### 2.2. Data Eligibility

The scientometric analysis was conducted using CiteSpace software (Version 6.1.R6 Advanced) [38]. Retrieved documents were imported into the software. A total of 27,007 cited references were automatically identified. Of them, 26,498 (98.12%) were valid and eligible for analysis. To be considered valid, documents must have had all seven criteria: author, title, year of publication, volume, pages, source, and DOI [39]. The data loss in the current study (1.88% of the total references) is acceptable, as it is within a typical range of 1.00–5.00% [40].

### 2.3. Document Co-Citation Analysis (DCA)

DCA is a type of scientometric analysis that creates a network of co-cited documents. DCA reveals main research trends in a specific topic of interest. The premise underlying DCA is that publications that are frequently cited together (i.e., co-cited) represent research clusters with a shared thematic interest [38,41,42,43]. Research clusters are built considering two types of nodes: citing and cited documents. DCA is based on the principles of graph theory. In the analysis, cited documents are modeled as the network’s nodes and co-citations as the network’s edges. Edge weights are assigned based on co-citation frequencies between documents [43]. DCA is conducted in CiteSpace by selecting “Reference” as Node Type on the main page of the software.

**Figure 1:**
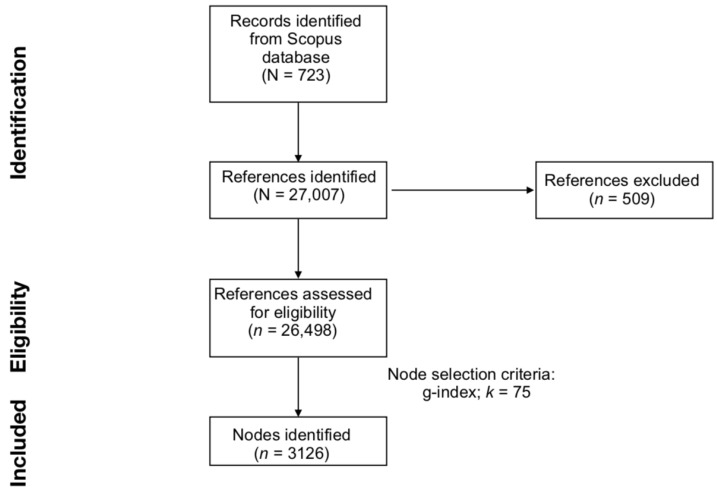
Preferred Reporting Items for Systematic Reviews and Meta-Analyses (PRISMA) flowchart of literature search, evaluation steps, and network generation.

To create a balanced network of clusters, CiteSpace allows adjustment of node selection criteria and scaling factors. The most common node selection criteria are the *g*-index, TOP *N*, and TOP *N%*. Node selection criteria set the strategy used to identify the main documents to include in the final network. The *g*-index is a derivative of the *h*-index, and it reflects the number of citations of an author’s top publications [39,44,45]. TOP *N* and *TOP N%* select and include in the final network the *N* and the *N%* most-cited items over a time interval, which, in this study, was fixed at 1 year. Node selection criteria are associated with scaling factors, which allow for controlling the size of the final DCA network. In the current study, the effects of multiple node selection criteria and scaling factors were compared to identify the optimal DCA network. As in previous publications (e.g., [46]), we selected the optimal DCA by comparing results generated with the *g*-index with scale factor *k* set at 25, 50, 75, and 100; TOP *N* with *N* set at 50; and TOP *N%* with N set at 10. The optimal node selection criterion and scaling factor were selected based on the number of nodes, number of major clusters, modularity (i.e., the degree to which a network is divisible into clusters), and mean silhouette score (i.e., the degree of homogeneity within individual clusters). Ultimately, the *g*-index with a scale factor *k* = 75 was selected to generate the DCA network. Figure 1 summarizes the main steps of the current work, from data collection to data analysis.

### 2.4. Metrics

Results are presented using structural and temporal metrics. Modularity Q, silhouette, and betweenness centrality are structural metrics. Modularity Q determines the level of divisibility of the network into separate modules or clusters. This metric varies between 0 and 1, with higher values indicating a more organized and divisible network [47]. Silhouette evaluates the internal homogeneity of a single cluster and how separate it is from other clusters in the network. Silhouette can have values between −1 and 1, where 1 represents the highest degree of internal consistency and separation from other clusters [48]. Finally, betweenness centrality measures how each node functions as a link between two other randomly selected nodes [49,50]. Values of betweenness centrality range from 0 to 1, with higher values indicating influential and impactful publications [51]. Temporal metrics include citation burstness and sigma. Citation burstness is computed with Kleinberg’s algorithm and signals a steep increase in the number of citations received by a document [52]. Finally, sigma combines betweenness centrality and citation burstness through the equation (centrality + 1)${}^{burstness}$ and indicates the level of influence and impact of a single document in the network [53].

## 3. Results

### 3.1. Bibliometric Analysis of the Citing Documents

The bibliometric analysis of the citing references revealed that the sample of 723 articles developed with an annual growth rate of 1.22% from 1962 to 2022. On average, each document received 31.24 citations.

The main sources of documents were *Infant Behavior and Development* (number of documents = 24), *Pediatrics* (number of documents = 17), and *Child Development* (number of documents = 15).

When considering authors’ affiliation country, the most frequent country that appeared in the sample was the United States of America (number of documents = 162; frequency = 0.2852—appearing in both single country publications, (SCP) = 150, and multiple country publications, (MCP) = 12—followed by Italy (number of documents = 39; frequency = 0.0687; SCP = 24; MCP = 15), and United Kingdom (number of documents = 34; frequency = 0.0599; SCP = 27; MCP = 7).

In the data pool, a total of 2656 distinct authors were found. Overall, an average of 0.272 documents per author were published, with each document having an average of 4.5 authors. The most productive authors in the sample were MH van IJzendoorn (number of documents = 16), BM Lester (number of documents = 14), K Michelsson (number of documents = 14), MJ Bakermans-Kranenburg (number of documents = 12), MH Bornstein (number of documents = 12), G Esposito (number of documents = 11), EM Leerkes (number of documents = 11), C Manfredi (number of documents = 11), CA Reyes-García (number of documents = 11), and LC Mayes (number of documents = 9).

The most frequently cited documents were authored by Prechtl [54] (total citations = 823) and Bell and Ainsworth [55] (total citations = 551).

**Figure 2:**
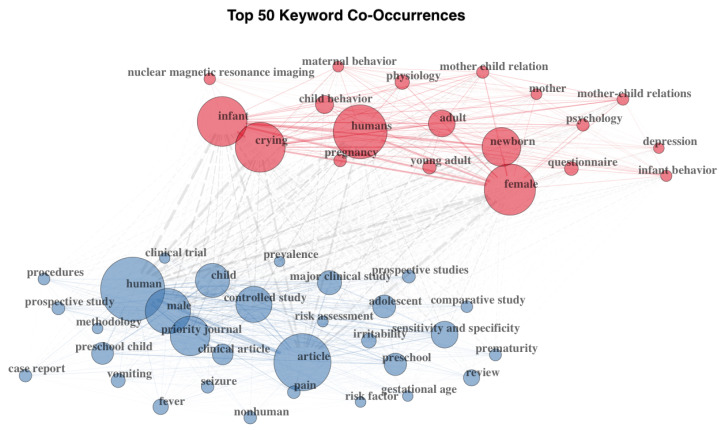
Top 50 keywords’ co-occurrences. Two clusters of co-occurring keywords were automatically identified based on the co-occurrence frequency. The two clusters are distinguished in the figure by color.

The collected documents included 1660 keywords. The most frequent keywords were *crying* (number of documents = 40), *infant crying* (number of documents = 35), *infant* (number of documents = 33), *infant cry* (number of documents = 29), *pain* (number of documents = 23), *cry analysis* (number of documents = 21), *infants* (number of documents = 21), *maternal sensitivity* (number of documents = 21), *cry* (number of documents = 18), and *parenting* (number of documents = 17; see Figure 2).

### 3.2. Structural Properties of the DCA Network

**Figure 3:**
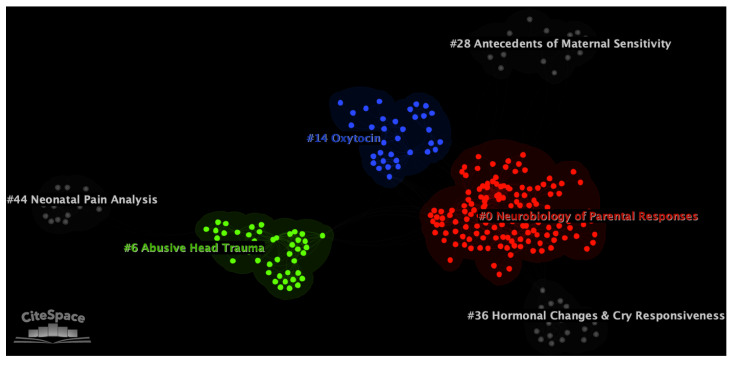
Document co-citation analysis network of the literature on infant cry, generated with CiteSpace software [38]. In the network, single nodes represent individual documents. Nodes are grouped into six main thematic clusters, which are depicted by color in the image. All clusters are identified by their IDs. Smaller IDs correspond to larger clusters. All clusters were manually renamed to reflect their content.

The final DCA network consists of 3126 nodes (i.e., documents) and 9300 links, with an average of 2.975 links for each node. The modularity Q value of the network is 0.978, and the weighted mean silhouette score is 0.961. These metrics indicate that the network of documents is divisible into distinct and homogeneous thematic clusters of research (see Figure 3).

In the network, six major thematic clusters were selected by the software based on their metrics. Clusters are labeled with the CiteSpace’s Log-Likelihood Ratio (LLR) algorithm. LLR was chosen because it provides the most accurate labels across automated labeling methods in CiteSpace [36]. The main clusters were automatically labeled as “Infant monitoring” (cluster #0; number of documents in the cluster (i.e., size) = 142; silhouette = 0.929; average year of publication = 2011), “Preventive Abusive Head Trauma” (cluster #6; size = 45; silhouette = 0.998; average year of publication = 2007), “Amygdala insula” (cluster #14; size = 35; silhouette = 0.994; average year of publication = 2009), “Infants monitoring” (cluster #28; size = 23; silhouette = 0.991; average year of publication = 2010), “Vasopressin concentration” (cluster #36; size = 20; silhouette = 0.996; average year of publication = 2016), and “Validation” (cluster #44; size = 15; silhouette = 0.983; average year of publication = 2002). The accuracy of all labels was subsequently confirmed by visual inspection of clusters contents. When the LLR label resulted in poor accuracy, the cluster was manually relabeled to reflect its thematic core (as, for instance, in Bonacina et al. [46]). Table 1 reports the metrics for the six major clusters in the network.

### 3.3. Impactful Documents

In the final network, a total of 10 documents had a citation burst in their history when setting γ at 0.60. The parameter γ represents the sensitivity threshold to identify citation bursts. The document with the strongest burst index was authored by Barr [56], with a value of 4.72 (burst duration = 7 years). In this work, the author described the infant crying curve, a behavioral function that defines the common developmental course of infant crying as an early increase that has its peak in the second month and then decreases until the fourth month. After this period, changes are minimal. The author also stressed the importance of studying developmental changes of infant crying in natural contexts. The following documents in terms of burst were authored by Strathearn et al. [57] (strength = 4.23; burst duration = 5 years) and by Swain et al. [58] (strength = 3.79; burst duration = 1 year). Barr [56] also showed the burst with the longest duration. Another document that reported the longest citation burst was authored by Atzil et al. [59] (burst duration = 7 years; strength = 2.80). Table 2 reports the main metrics for the ten most impactful documents in the network.

## 4. Discussion

The scientific content of the major thematic clusters of research is examined in this discussion. A qualitative investigation of the main documents included in clusters is conducted in this section. Clusters are presented in ascending chronological order of the average year in which documents in the cluster were published. For the major citing documents, their coverage (i.e., the number of documents in the cluster that were cited by the paper) and their global citation score (GCS; i.e., the total number of citations received by the publication in Scopus) are provided.

### 4.1. Cluster #44: Neonatal Pain Analysis

The earliest major cluster to appear in the literature is cluster #44, which included documents whose average year of publication was 2002. The major citing document was authored by Sisto et al. [65] (coverage = 13; GCS = 17). This document tests the reliability of a new automated approach to cry analysis (i.e., ABC analyzer) based on the ABC scale [66]. The ABC analyzer relies on three acoustic parameters: pitch frequency, amplitude, and presence of “siren cry” (i.e., a specific frequency- and amplitude-modulated cry feature). The authors compared the ABC analyzer to the results obtained with the Douleur Aigue du Nouveau-Né scale [67], a validated scale for pain measurement. Importantly, the authors found that results obtained by means of the two methods aligned. The article by Sisto et al. [65] stemmed from several documents that investigated the possibility of an objective linkage between cry acoustic features and pain level analysis [68,69,70]. In a similar vein, Bellieni et al. [71] found a correlation between different pain intensity levels and specific crying patterns in full-term neonates. This research revealed a relation between Douleur Aigue du Nouveau-Né scale scores higher than 8 and emission of the “siren cry” in infants. When pain crossed this value, crying started with a high pitch followed by the “siren cry”, and volume was retained at its peak. The identification of a relation between acoustic patterns of crying and pain has clinical implications, as it alerts caregivers to the need for timely pain management in newborns [72]. Additionally, because pediatric pain and distress measurement in neonates are largely influenced by the interpretation of crying signals, the healthcare sector would benefit considerably from an objective evaluation of infant crying [68,72,73].

### 4.2. Cluster #6: Abusive Head Trauma

Cluster #6 includes documents published on average in 2007. The major citing documents in the cluster were authored by Barr [25] (coverage = 15; GCS = 83), Douglas et al. [74] (coverage = 11; GCS = 28), and Lopes et al. [75] (coverage = 9; GCS = 44). Abusive head trauma and shaken baby syndrome are terms referring to all symptoms and consequences due to voluntarily shaking or hitting the infant’s head [25]. This type of abuse can result in subdural hematoma, cerebral edema, and retinal hemorrhage, damage that can lead to severe long-term consequences and, in some cases, to death [76,77]. In the U.S., approximately 1300 abusive head trauma cases are reported annually, with a fatality rate of 25% [78]. Several articles in the cluster identify frequent and unsoothable crying as a central trigger for this conduct [25,79,80]. The early increase in crying around the first months of life can threaten the caregiver–infant relationship, as it is perceived by caregivers as something wrong with the infant or with the caregiver’s ability to soothe the baby [25,81]. This is because the baby’s crying often does not have an easily recognizable cause and the normal peak of the amount of crying is interpreted by parents as a clinical condition [25,74]. These traits that characterize infant crying at this age are considered precipitating for abusive head trauma, and the scientific literature summarized them with the clinical label of “colic syndrome” [76,82]. Starting from these premises, most articles in the cluster focus on prevention activities that aim to spread more accurate knowledge about the normal development of infants, their crying, and the phenomenon of abusive head trauma [25,75,81,83,84]. Prevention activities allow caregivers to reduce their concern about excessive infant crying and promote optimal behavioral strategies in the case of excessive frustration [81,84]. Common and effective advice is to walk away or leave the room if frustrated by an infant’s cry [81,84]. The effectiveness of educational programs was tested through the use of Period of PURPLE Crying prevention materials in North America and in Japan, a video-based program developed to inform parents about infant crying and safe parenting. An intervention group reported higher scores on crying knowledge scales, percentage of sharing of advice to walk away if frustrated by crying, and in the percentage who walked away during inconsolable bouts of crying [81,85].

### 4.3. Cluster #14: Oxytocin

The third major cluster in terms of average year of publication is cluster #14, which includes papers published on average in 2009. The major citing documents in the cluster were authored by Riem et al. [86] (coverage = 11; GCS = 274), Mah et al. [87] (coverage = 9; GCS = 25), and Feldman and Vengrober [88] (coverage = 7; GCS = 115). The central topic of the cluster is the role of oxytocin in caregiver response to infant cry. Overall, oxytocin promotes attachment and maternal behavior in many animals [89]. Among mammals, oxytocin is crucial to various aspects of motherhood, such as parturition, lactation, and engaging in parenting and social interactions with offspring [90]. For instance, in rats, when regions with a high density of oxytocin receptors (e.g., periaqueductal central gray matter) are pharmacologically or physically inhibited, maternal behaviors are suppressed [91]. In humans, for instance, oxytocin seems to play a role in maternal perception of the urgency for intervention when babies cry [87]. Mah et al. [87] tested and compared the results of nasal administrations of oxytocin and placebo on sensitive caregiving in mothers with postnatal depression. After intranasal administration of oxytocin, as compared with a placebo, mothers rated prerecorded cry sounds as more urgent and would choose a rapid intervention. The role of oxytocin in facilitating caregiving behaviors has also been studied in regard to hostile parenting. Bakermans-Kranenburg et al. [92] showed how oxytocin administration decreases the use of excessive handgrip force in women when listening to infant cries, especially for women with a history of harsh discipline in childhood. Another study that marked the effects of oxytocin on parental responsiveness was conducted by Riem et al. [86]. Here, the authors assessed neural responses to infant crying using functional magnetic resonance imaging to compare a group of women who were administered oxytocin and a placebo group. The authors observed reduced activation in the amygdala and increased activation in the insula and in the inferior frontal gyrus pars triangularis in the group of women who were administered oxytocin, as compared to the placebo group. These results suggest that oxytocin provokes a more responsive reaction to infant crying by decreasing activity in neural circuits associated with anxiety and aversion, and by increasing activity in brain regions involved in empathy.

### 4.4. Cluster #28: Antecedents of Maternal Sensitivity

Cluster #28 consists of documents published on average in 2010. The major citing documents in the cluster were authored by Biro et al. [93] (coverage = 9; GCS = 10), Leerkes et al. [94] (coverage = 5; GCS = 69), and Leerkes et al. [95] (coverage = 4; GCS = 49). Documents included in the cluster focused on examining predictors of maternal sensitivity to infant signals. Maternal sensitivity is defined as the mother’s ability to perceive and interpret accurately her own infant’s cues and act appropriately in response to them [93,94,96,97,98]. Overall, mothers with strong and secure attachments seem to be more capable of noticing their infants’ cues, interpreting them positively, and regulating their own emotions in an adaptive way. Altogether, these factors contribute to ensuring higher maternal sensitivity [94,97,99]. For some authors, maternal sensitivity is made possible because the mother’s attachment representation influences the way in which she perceives, interprets, and responds to her infant’s distress signals [94,97,99]. Constructs such as reflective ability, maternal insightfulness, and attribution of meaning about infant behaviors and emotions influence establishment of the attachment bond [100]. The important role of these factors in maternal sensitiveness is supported by Dykas and Cassidy [99], who observed that individuals with secure internal working models of attachment are more likely to process social information positively and to utilize the knowledge derived from their positive attachment experience to interpret information. A similar perspective was proposed by Ablow et al. [97], who measured respiratory sinus arrhythmia, skin conductance levels, and heart rate in first-time expectant women in response to infant cry. In the literature, these parameters are known to capture immediate physiological responses to stressors and can be used as predictors of sensitivity [94,95,97]. In response to cry, secure–autonomous women showed a decline in respiratory sinus arrhythmia, reflecting approach-oriented responses. In contrast, insecure–dismissing women exhibited increased respiratory sinus arrhythmia and an electrodermal response, reflecting a behavioral inhibition that is more likely to be maladaptive in parenting. Insecure–dismissing women rated infant cries as more aversive than secure–autonomous women [95,97].

### 4.5. Cluster #0: Neurobiology of Parental Responses

The average year of publication of documents included in cluster #0 was 2011. The major citing documents in the cluster were authored by Bornstein [101] (coverage = 17; GCS = 0) and Kim et al. [102] (coverage = 13; GCS = 36). The main topic of the cluster’s documents is the investigation of the neurobiological mechanisms concerning parental responses [1]. In this selection of the literature, several neuroimaging studies were conducted to explore the underpinnings of parental responses while parents were exposed to infant stimuli. These studies reported neural activation in the putative mirror neuron system, the anterior insula, thalamo-cingulate regions, and the dorsomedial prefrontal cortex, which are involved in the comprehension of others’ facial expressions, others’ feelings, and others’ thoughts [58,102,103]. Similarly, parents exposed to infant stimuli show enhanced neural activity in the ventral tegmental area, substantia nigra, ventral striatum, and medial orbitofrontal cortex. These regions are part of the reward system involved in approach-related motivation. Furthermore, enhanced brain activity was observed in the supplementary motor area and premotor cortex, which control the preparation of motor responses [58,102,103]. Finally, the lateral prefrontal cortex, which is pivotal for emotion regulation systems, emerged as involved in parental responses [58,102,103].

At the neural level, some gender differences in detecting and responding to infant cry stimuli were documented. After the exposure to infant crying cues, De Pisapia et al. [10] found that the dorsal medial prefrontal and posterior cingulate areas remain engaged in men, regardless of their parental status. Conversely, in women, these areas display decreased activation. Furthermore, the brains of adult females seem to be prepared to automatically trigger motor responses when they hear the cries of infants [104]. Despite the differences in the activation of neural circuits, research suggests that mothers’ and fathers’ brains show synchronous patterns of activity in regions involved in mentalization and empathy when exposed to infant cues, such as infant vocalization [6].

### 4.6. Cluster #36: Hormonal Changes & Cry Responsiveness

In cluster #36, the documents were published, on average, in 2016. The major citing documents in the cluster were authored by Rybicka et al. [105] (coverage = 10; GCS = 3) and Kaźmierczak et al. [106] (coverage = 10; GCS = 1). Several citing and cited documents in the cluster investigated the role of oxytocin and vasopressin in parental care and their regulation at the genetic level [105,106,107,108,109]. Apter-Levi et al. [108] hypothesized that oxytocin and vasopressin support distinct patterns of parent–infant interaction. In fact, parents with high oxytocin levels, as compared to parents with low oxytocin levels, showed more affectionate engagement and salience for social inputs. Overall, G-carriers in the rs53576 polymorphism on the oxytocin receptor gene show greater heart rate responses to infant cries and optimal neural responses in terms of promptness to action [86,110,111]. Additionally, parents with high vasopressin tend to engage in stimulatory contact and to increase the object-salience when infants show a desire for social connection [108]. Overall, vasopressin seems to influence paternal more strongly than maternal parenting [112]. Studies showed that vasopressin increases social recognition in both animals and humans and plays a role in male bonding and territorial behaviors in animals [108,112]. Thus, vasopressin could support fathers’ capacity to discern others’ intentions and protect the mothers and offspring. Accordingly, Leerkes et al. [113] showed that carriers of long alleles in the arginine vasopressin receptor 1A tend to show negative beliefs and attributions about their infant crying and, consequently, less sensitivity when responding to it.

### 4.7. Study Limitations

Scientometric analysis provides insight into the literature on infant crying and caregiver responsiveness. However, this approach to deconstructing the literature has some intrinsic limitations. Two factors that might alter the outcomes of scientometric analysis are the chosen search string and the platform for conducting the literature search. It is, therefore, possible that some relevant documents in the field were excluded because the terminology in their title, abstract, and keywords eluded the search string. Similarly, some documents might have been excluded because they were published in journals that are not indexed in Scopus. However, the current work was based on analysis conducted on a large sample of 723 documents, as well as the 26,498 references that they cite. Another limitation is that the scientometric approach relies heavily on quantitative relationships between documents, without providing insight into the qualitative relationships. Frequent co-citation does not necessarily indicate the value of documents. In fact, certain publications may receive many citations because they are innovative and impactful or because they receive considerable criticism from works in the same field. For this reason, in this analysis, we inspected clusters qualitatively to identify research trends [114].

## 5. Conclusions

In the current study, the literature on infant crying and caregiver responsiveness was reviewed using a scientometric approach. In doing so, the scientometric approach proved useful in illuminating the main research themes and most impactful publications in the literature on infant cry and caregiver responsiveness. In particular, current trends prioritize exploration of the neurobiology of caregiver responses and the identification of antecedents of maternal sensitivity, such as hormonal levels and attachment quality. Likewise, the literature strongly focused on mothers and their responsiveness to infant cry. In this trend, the role of fathers was only marginally investigated. Research on paternal responsiveness to infant cry represents material for future lines of research that aim to provide insight into the neurobiology of fatherhood. Similarly, the ways in which the quality of the relationship between caregivers influences their responsiveness to infant cry are still poorly understood. In the literature, abusive head trauma has been found to correlate with a poor understanding of the typical development of infant crying but also with a lack of effective strategies to stem crying-related frustration and effective ways to console babies. In this context, recent studies have developed evidence-based strategies for soothing crying babies and preventing maladaptive responses in caregivers. Understanding the connections among infant crying and caregivers’ mental health, parenting practices, and cultural beliefs can help design evidence-based interventions to promote positive and responsive caregiving and reduce the risk of adverse outcomes for both infants and their caregivers.

## Figures and Tables

**Table 1:** Details of the six major thematic clusters identified using document co-citation analysis. Cluster ID refers to the ID of the identified cluster. Size reflects the number of documents included in a cluster. Cluster IDs and sizes are inversely related. Silhouette score varies between 0 and 1 and represents the degree of homogeneity of a cluster. Mean Publication Year represents the average year in which documents included in a cluster were published. Log-Likelihood Ratio (LLR) label was automatically generated by CiteSpace software to name the clusters. An alternative label (Suggested Label) is provided after a qualitative inspection to reflect the content of clusters.

Cluster ID	Size	Silhouette	Mean Publication Year	LLR Label	Suggested Label
0	142	0.929	2011	Infant monitoring	Neurobiology of Parental Responses
6	45	0.998	2007	Preventing Abusive Head Trauma	Abusive Head Trauma
14	35	0.994	2009	Amygdala insula	Oxytocin
28	23	0.991	2010	Infants monitoring	Antecedents of Maternal Sensitivity
36	20	0.996	2016	Vasopressin concentration	Hormonal Changes & Cry Responsiveness
44	15	0.983	2002	Validation	Neonatal Pain Analysis

**Table 2:** Main metrics of the top ten citation burst documents.

Reference	Citation Burstness	Publication Year	Burst Began	Burst Ended	Burst Duration (Years)
Barr [56]	4.720	1990	1991	1998	7
Strathearn et al. [57]	4.234	2008	2011	2016	5
Swain et al. [58]	3.789	2014	2016	2017	1
De Pisapia et al. [10]	3.157	2013	2016	2017	1
Barrett and Fleming [60]	2.993	2011	2015	2017	2
Atzil et al. [59]	2.801	2011	2012	2019	7
Abou-Abbas et al. [61].	2.666	2015	2017	2022	5
LaGasse et al. [62]	2.546	2005	2012	2013	1
Musser et al. [63]	2.540	2012	2015	2016	1
Barr et al. [64]	2.492	1988	1994	1996	2

## Data Availability

Not applicable.

## Abbreviations

The following abbreviations are used in this manuscript:

ASD	Autism Spectrum Disorder
DCA	Document Co-Citation Analysis
GCS	Global Citing Score
LLR	Log-Likelihood Ratio
MCP	Multiple Country Publications
PRISMA	Preferred Reporting Items for Systematic Reviews and Meta-Analyses
SCP	Single Country Publications
